# Expertise-dependent mental representation in chess: evaluation and comparisons based on structural dimensional analysis-motoric

**DOI:** 10.3389/fpsyg.2026.1695175

**Published:** 2026-03-13

**Authors:** Thomas Küchelmann, Konstantinos Velentzas, Christian Schütz, Thomas Schack

**Affiliations:** 1 Department of Neurocognition and Action-Biomechanics, Faculty of Sport Sciences, Bielefeld University, Bielefeld, Germany; 2 Center of Excellence Cognitive Interaction Technology – CITEC, Bielefeld, Germany; 3 Department of Physiotherapy, Fulda University of Applied Sciences, Fulda, Germany

**Keywords:** chess expertise, long-term memory, mental representation, structural dimension analysis of mental representation, task-related concept

## Abstract

**Introduction:**

Research findings underline that human behavior and decisive action significantly depend on knowledge accessibility in long-term memory (LTM). For this purpose, various methods have been conducted and applied to help researchers gain insights into LTM functioning. These methods are based on traditional low-cost instruments (e.g., think-aloud protocols, memory protocols, questionnaires, a. o.) as well as modern high-cost technologies.

**Method:**

Furthermore, an emerging method that evolves traditional research techniques in a digitalized environment is *Structural Dimensional Analysis-Motoric*. This analysis is based on participants’ preferences regarding the closeness of given task-related concepts (TRCs) during a sorting process. From this perspective, chess is a highly cognitive domain involving an immense amount of specific knowledge—a reason it became a prominent field in cognitive research. The present study aims to examine how strategy-related patterns (meaningful and interconnected standardized chess motifs) are incorporated into the LTM of chess players, depending on their expertise (novices, intermediates, and high-level players).

**Results:**

The analysis shows a significant similarity between experts and intermediate chess players but no significant results for the comparisons between experts and intermediates to novices.

**Discussion:**

Researchers should make efforts to expand mental representation research in chess, for example, by manipulating a variety of strategy-related patterns (e.g., critical openings, middle games, and endgame situations) and/or enhancing the difficulty of the TRCs. The results can be applied to the further development of augmented feedback (e.g., assistive training systems) and virtual players (platforms).

## Introduction

Knowledge organization and processing is a key focus in both modern cognitive and sport sciences. Many theories have been proposed to address the fundamental question of how knowledge (e.g., events, objects, relationships, schemas) is structured and stored in human memory. The concept of memory incorporates the categorizations of short-term memory (STM), long-term memory (LTM), and working memory (WM; Cowan, 2008). The storage of information for a limited period of time is defined as STM (Cowan, 2008). LTM is consistently interpreted as a vast portfolio of knowledge, also known as mental representation, about past events, which undoubtedly constitutes a functioning human being (Cowan, 2008). Finally, the term WM was first introduced by Miller et al. (1960) and includes mechanisms for activation, control, and active maintenance of task-relevant information for the performance of complex actions requiring high cognition (Miyake and Shah, 1999).

From a methodological point of view, researchers have employed various approaches to assess information stored in LTM. Firstly, traditional low-cost methods such as think-aloud protocols (e.g., De Groot, 1946/1966), memory protocols (e.g., Chase and Simon, 1973a,b), questionnaires, and paper-pencil tests (e.g., D’iakov, 1927) have been applied to assess, on the one hand, quantitative differences in chess-specific knowledge stored in LTM and, on the other, cognitive abilities like attention and imagination. Secondly, to investigate expertise-dependent changes in brain structures or brain activation patterns, modern high-cost technologies have been used. These technologies provide a significant increase in objectivity and reliability but offer less usability. Examples include Magnetoencephalography (MEG, e.g., Amidzic et al., 2001), functional Magnetic Resonance Imaging (fMRI, e.g., Bilalić et al., 2011), Positron Emission Tomography (PET, e.g., Nichelli et al., 1994), and Single Photon Emission Computed Tomography (SPECT, e.g., Onofrj et al., 1995).

A substantial body of chess studies has been conducted since the seminal study of De Groot (1966), with the aim to elucidate cognitive strategies that enable chess experts to outperform less-skilled players in chess-specific memorization tasks and in the retrieval of chess knowledge (e.g., Chase and Simon, 1973a; Gobet and Simon, 1996; Simon and Gilmartin, 1973). The results of these studies suggest that *chunking* is the *hidden strategy* that, on the one hand, reduces cognitive load through clustering and, on the other, enhances the accessibility of chess knowledge. For instance, Simon (1996) presented a considerable amount of empirical and model-based evidence suggesting that experts store between 50,000 and 1.8 million chunks in their LTM. In summary, the results of De Groot (1946/1966, 1978), Chase and Simon (1973a), Simon and Gilmartin (1973), Gobet and Simon (1996), and Simon (1996) led researchers to hypothesize that high-level chess expertise involves learning a vast vocabulary of chunks through experience and deliberate practice, which facilitates fast recognition and retrieval processes (i.e., the chunking theory). This learning process is akin to acquiring a language vocabulary (e.g., Barking, 2024). A well-structured vocabulary facilitates easy retrieval and application within an overarching context, such as grammatical rules, along with various estimates and simulations regarding its volume (in the context of chess, e.g., Simon and Gilmartin, 1973). However, learning grammatical rules and a large vocabulary of words is not sufficient for speaking a language. It is important to organize the words that the vocabulary consists of into concepts and categories in LTM, thereby building a mental representation of the vocabulary. This mental representation comprises the clustering of words (i.e., words that belong to the same category are stored in the same corresponding cluster). Therefore, in the present study, we investigate and analyze how chess players of three different skill levels (i.e., experts, intermediates, and novices) have organized specific strategy-related patterns into clusters within their mental representation.

As with all scientific methods, which have their pros and cons, some of the aforementioned low-cost methods are contested and criticized (Thomas and Thomas, 1994). For example, think-aloud protocols, which are widely applied in the cognitive sciences to deliver valid and unadulterated information, are often questioned due to the time-consuming analytical process. On the other hand, the high-cost methodologies previously mentioned are characterized by their limited usability, due to the need for immobile, large, space-consuming, and/or expensive equipment. This raises questions about both scientific principles and practical applications. The scientific question involves the use of various types of evidence in a convergent manner, while the application question seeks to determine the most effective convergent method for a particular application, taking into account time and cost factors.

One of the most recently developed research methods, which allows for an effective and differentiated statistical comparison of knowledge representation both in individuals and groups, and that facilitates a simple visualization of clusterings in mental representations, is *Structural Dimensional Analysis* (*SDA*). This method was first developed in the fields of linguistics and cognitive psychology (Lander and Lange, 1996), based on Symbolic Learning Theory (Sackett, 1934) and Schema Theory (Anderson, 1977). Secondly, in order to analyze and compare motor programmes in sports sciences, Schack (2002) adapted and enhanced the usability of SDA by implementing and automating the method in a computer environment (software named *Structural Dimensional Analysis-Motoric*, *SDA-M*). A significant advantage of SDA-M is its time efficiency and automated statistical analysis. SDA-M provides insights into the participants’ mental representations and allows for statistical comparisons between groups and individuals (e.g., cluster invariance) in LTM, which offers insights into the chunking process (Eysenck and Keane, 2005). From this point of view, SDA-M is not only applied in cognitive and sports sciences (Schack, 2002, 2004, 2010) but also in the sport psychological assessment of regulatory strategies (Velentzas et al., 2010, 2011) and in medical and rehabilitation diagnostics (Braun et al., 2007). A substantial body of research employing SDA-M supports the notion that participants’ mental representation structures are distinctly associated with their skill-level (Lander and Lange, 1996; Schack and Mechsner, 2006). This notion, regarding differences in the mental representation of domain-specific knowledge depending on expertise, aligns perfectly with findings from chess research. For instance, the idea that expertise-dependent chunking reduces cognitive costs and aids in the retrieval of chess knowledge (Chase and Simon, 1973a; Gobet and Simon, 1996; Simon and Gilmartin, 1973). The influence of expertise on how practitioners’ mental representations differ in specific knowledge areas has been studied using SDA-M across various subjects, for instance in dancing, volleyball, climbing, and handball (Bläsing et al., 2009; Velentzas et al., 2010; Bläsing et al., 2014; Vogel and Schack, 2023), for the evaluation of psychological strategies (Velentzas et al., 2011), and of manual everyday actions and manual assembly procedures (Strenge and Schack, 2021). All of these studies show, on the one hand, that the mental representations of experts are more functionally structured compared to those of less-skilled participants. Furthermore, intervention studies have demonstrated that explicit training leads to a meaningful reorganization of participants’ mental representations, improving their similarity to those of experts (Hennig et al., 2017; Velentzas et al., 2011).

Taking this into account, it is of significant interest to gain insights into how chess-specific knowledge is organized in LTM (i.e., statistical analysis of clustering), and SDA-M offers this opportunity. The present study aims to bridge this gap adapting SDA-M to the chess environment. It investigates chess players’ mental representation of fundamental chess tactics, with a special focus on expertise. Through the adaptation of SDA-M in chess, we refer to *task-related concepts* (*TRCs*) when discussing chess tactics selected for the study. Based on established research findings concerning mental representations and cognitive psychological chess expertise (e.g., Chase and Simon, 1973a; Gobet and Simon, 1996; Simon and Gilmartin, 1973), we hypothesize that experts will exhibit a more functional structure in the sense of clustering of represented knowledge in their mental representation, compared to the other groups involved in the study. We expect that experts will reveal knowledge clusters implying similar TRCs, whereas non-experts will provide a less meaningful clustering.

## Materials and methods

### Study design

In the present study, we used the level of expertise as the independent variable and the invariance values (i.e., comparisons between experts’ mental representations and those of other groups) as the dependent variable. In line with existing manipulation checks and in order to avoid cognitive overload during the data collection, we define in total the number of 15 TRCs which can be assigned into five fundamental chess strategy-related patterns. The selection of the TRCs was based on pattern recognition tasks, which are an integral part of basic chess training (e.g., Colditz, 2016; Neistadt, 1997) and planning tactics (Krogius et al., 1980; Tarrasch, 1931).

For the data collection, a desktop with Windows 10 (64-bit operating system, x64-based processor Intel(R) Pentium(R) Silver N5030 CPU 1.10GHz) and an LCD HD screen (13.5 inches, 100 Hz) was used. Data collection was performed by trained assistants. Before data acquisition, all participants adjusted their chair distance to the screen (50 cm) to ensure a clear view of the stimuli. Each individual session, including 15 × 14 = 210 sorting comparisons, took between 30 and 40 min.

### Participants

A number of 51 volunteer chess players (*M*_age_ = 32.07 years, *SD*_age_ = 15.18 years) participated in the present study. They were recruited in collaboration with chess clubs across North Rhine-Westphalia and Hamburg. Only participants with uncorrected normal vision were accepted; individuals whose vision was corrected to normal with contact lenses or glasses were not eligible. The participants were assigned into three groups (experts, intermediates and novices), considering firstly their ELO/DWZ^1^ ratings (international ranking system, Elo, 1978) and secondly the self-reported chess experience in years. An ELO/DWZ score of 1,850 and above represents an expert (i.e., Class A player or better). An ELO or DWZ between 1,200 and 1,849 defines an intermediate player (i.e., Classes B to D). Participants with an ELO or DWZ of 0 to 1,199 (i.e., Class E and below) or who reported having played a maximum of 100 chess games were classified as novices. Fourteen participants were classified as experts (*M*_age_ *=* 34.43, *SD*_age_ *=* 13.12), 18 as intermediates (*M*_age_ *=* 38.55, *SD*_age_ *=* 16.41) and finally 19 as novices (*M*_age_ *=* 24.19, *SD*_age_ *=* 12.23). Prior to data acquisition, all participants provided informed consent in accordance with the Declaration of Helsinki and the ethical guidelines of the home university.

### Stimuli and procedure

**Figure 1:**
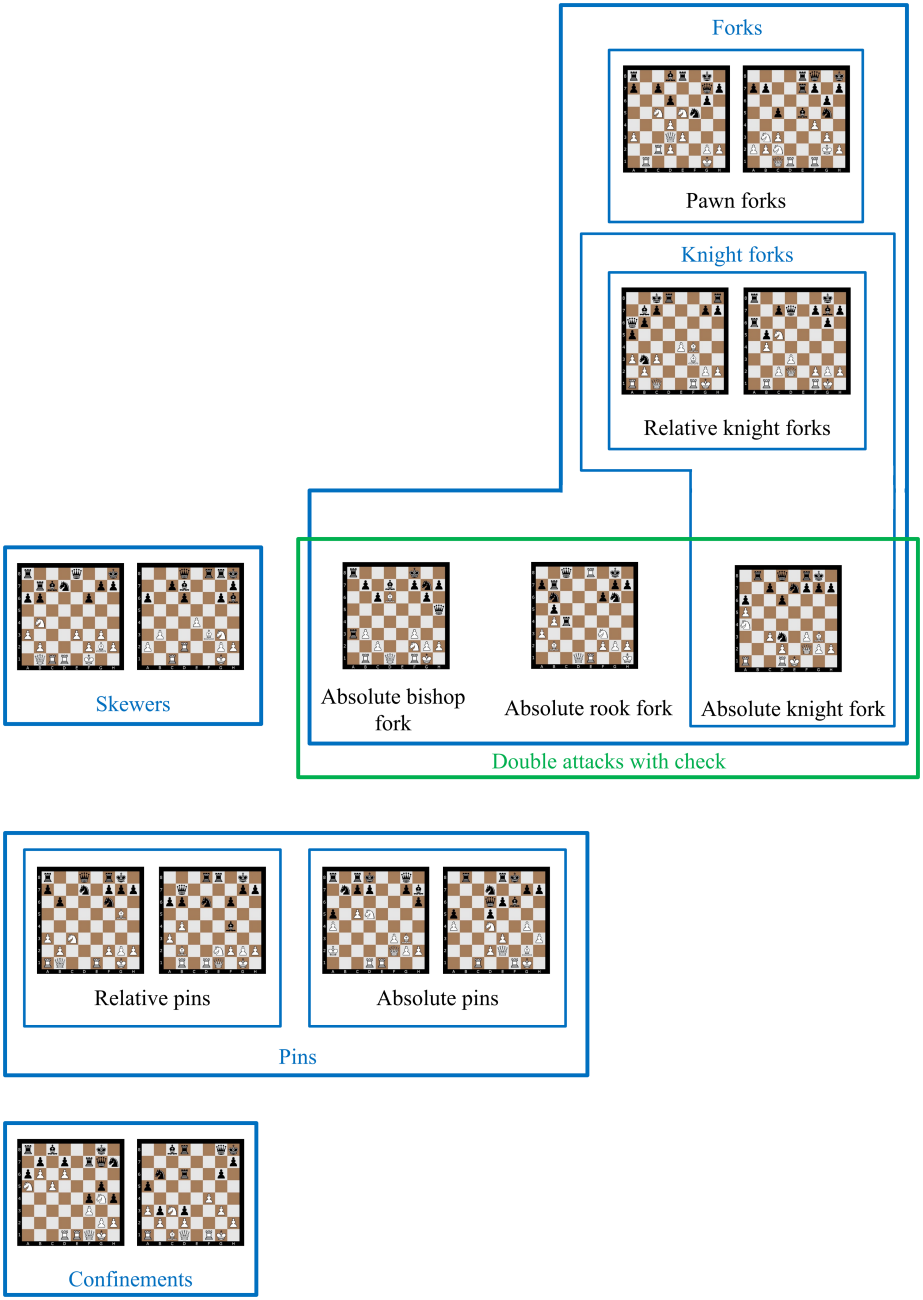
The stimuli, classified according to five overarching main concepts (forks, skewers, double attacks with check, pins, and confinements) and their subcategories.

All 15 stimuli are chess-TRCs displayed on full 8 × 8 chessboard diagrams in randomized order. They represent the following fundamental strategy-related patterns in chess (a) *forks* (i.e., a piece attacking multiple enemy pieces simultaneously), (b) *skewers* (i.e., a line piece such as a bishop, rook, or queen attacking two enemy pieces in a line, with one piece, typically the more valuable, under direct attack and the less valuable piece placed behind it), (c) *double attacks with check* (i.e., simultaneously attacking the king and a high-value piece, such as a queen, rook, knight, or bishop), (d) *pins* (i.e., a defending piece cannot move out of the attacking piece’s line without exposing a more valuable defending piece), and (e) *confinements* (i.e., pieces unable to move because they are blocked by other pieces). These concepts are further classified into the following nine subcategories (Figure 1): (1) *pawn forks* (i.e., a fork executed by a pawn), (2) *relative knight forks* (i.e., a fork executed by a knight that does not check the king), (3) *absolute knight forks* (i.e., a fork executed by a knight that checks the king), (4) *absolute rook forks* (i.e., a fork executed by a rook that checks the king), (5) *absolute bishop forks* (i.e., a fork executed by a bishop that checks the king), (6) *skewers by a bishop*, (7) *relative pins* (i.e., a piece of greater value than the pinning piece, but not the king, is in the pinning piece’s line or diagonal), (8) *absolute pins* (i.e., the king is in the pinning piece’s line or diagonal), and (9) *confinements of bishops* (i.e., a bishop is stuck on the baseline).

**Figure 2:**
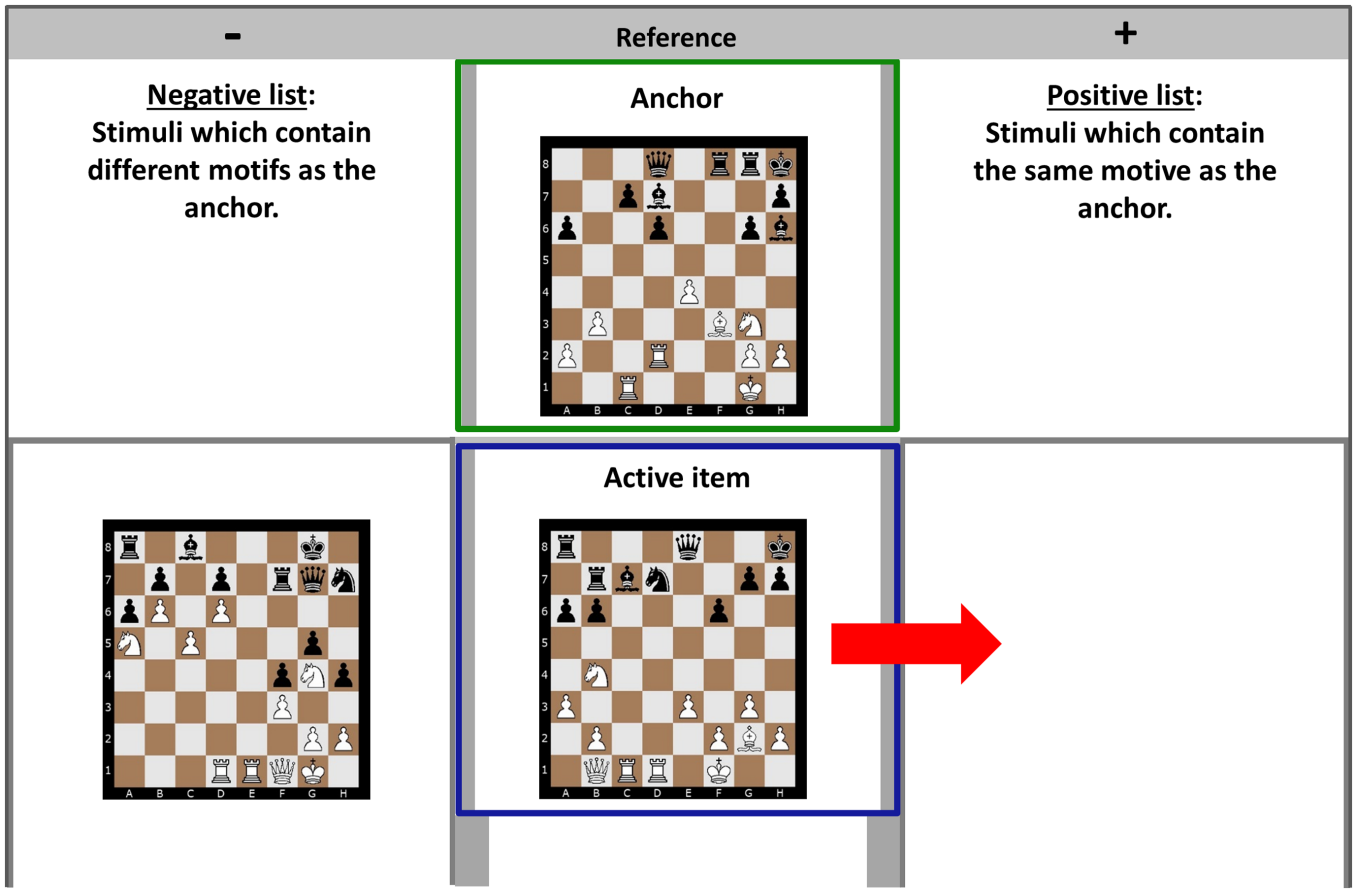
User interface of the splitting procedure: the active item displays a skewer (one of Black’s bishops attacks a rook, with a second rook positioned behind the first on the same diagonal) and is therefore similar to the anchor (i.e., the reference), which also displays a skewer. Consequently, the active item should be included in the positive list.

The data collection with SDA-M starts with a *split procedure* (Schack and Hackfort, 2007) that includes a multiple sorting/comparison task (Figure 2). The split procedure objectifies human memory structures of specific tasks by translating decision-making (i.e., comparisons of TRCs) into metrical binary data, which in subsequent analytical steps allows the illustration of the underlying mental representations for individuals and groups displayed as a tree diagram (Schack and Tenenbaum, 2004). Consequently, the number of comparisons depends on the number “*15*” of the selected TRCs (15^2^–15). During the split procedure, in randomized order, each TRC is presented as a point of reference (the so-called “anchor”), and the remaining 14 TRCs must be classified as “positive” (i.e., containing the same motif as the anchor) or “negative” (i.e., containing different motifs from the anchor) based on the hidden strategy-related pattern. Once all 14 TRCs are classified, another TRC is randomly placed as the anchor. The sorting process concludes when each TRC has been used as an anchor.

## Analytical process

The SDA-M implies a four-step analytical process, beginning with the sorting phase, followed by mathematical calculations, leading to the visualization of mental structures (e.g., clusters), and concluding with invariance comparisons between individuals and groups. The first step (the split procedure, as mentioned above) involves 15 × 14 (anchor × TRC) decisions/comparisons, generating a *decision matrix* for each participant. The subsequent step entails obtaining algebraic subtotals for each partial quantity across all decision matrices. To standardize these summarizing matrices, a *Z*-transformation is calculated, resulting in a so-called *Z-matrix*, which is used for the following analytical steps.

**Figure 3:**
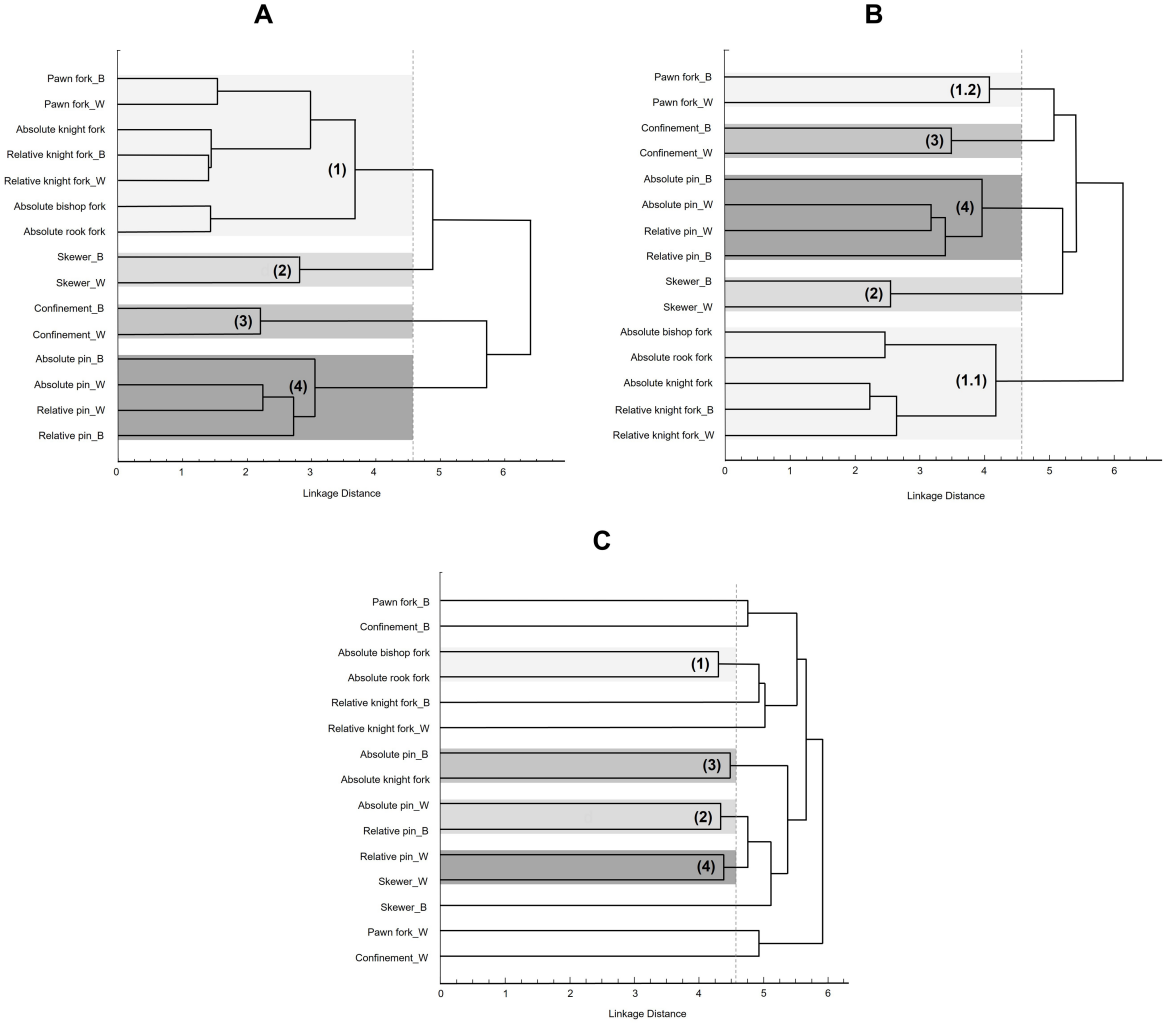
Group dendrograms of **(A)** all experts: (1) forks, (2) skewers, (3) confinements, (4) pins; **(B)** all intermediates: (1.1) forks without pawn forks, (1.2) pawn forks, (2) skewers, (3) confinements, (4) pins; **(C)** all novices: (1) absolute forks, (2) an absolute and a relative pin, (3) a fork and a pin, (4) a skewer and a pin.

Based, on the *Z*-matrix an Euclidean distance cluster analysis (Schack, 2012) generates the mental representations’ *tree diagrams* (Figure 3). In this tree diagram profile, the TRCs are linked or not to each other according to the analysis, building at the same time sub-clusters, whereas in the best case TRCs with similar strategy-related patterns are included in the same sub-clustering. Each of the cluster solutions is identified as having a critical Euclidean distance (Equation 1). According to the number *N* of TRCs and the chosen critical value for *α* (in our case *α* = 0.05), a threshold—*d_krit_*—is calculated by


$$d_{\text{krit}}=2N\cdot 1-r_{\text{krit}}\;(\alpha ,\mathrm{FG}\;|\;H_{0}:r=1)$$ (1)

In Equation (1), $r_{\text{krit}}$ denotes the randomness-critical correlation value. If the correlation value of two TRCs is less than $r_{\text{krit}}$ , then they differ significantly from each other. Clusters or sub-clusters underneath this critical value are perceived as statistically relevant for the next step of similarity comparison.

The so-called *invariance value λ* is calculated by considering (a) the number of significant cluster solutions and (b) the TRCs included in these clusters. For this purpose, the determined individual cluster solutions must be examined pairwise for their structural invariance, which is based on the *invariance matrix* (*λ*) in Equation (2).^2^ To calculate *λ*, it is necessary to compare the number of clusters formed by the pairwise cluster solutions. The number of TRCs within these clusters is the partial quantity *n*_i_ and *n*_k_ with 0 ≤ *i*, *k* ≤ *N*. For the structural invariance measure of two cluster solutions, the average size *n*_ik_ of the clusters formed is used. (*λ*) is determined by


$$\lambda_{\mathrm{ik}}=k_{\mathrm{ik}}\;\mathrm{GAM}(p_{\mathrm{ik}})$$ (2)

which involves the ratio of the number of clusters present between the test subjects.


$$k_{\mathrm{ik}}=\frac{\min(r,s)}{\max(r,s)}\;(\text{limiting value}\;k_{0}=\frac{2}{3})$$ (3)

where *r*, *s* is the number of clusters contained in both cluster solutions in Equation (3), and *GAM(p_ik_)* in Equation (2) is the weighted arithmetic mean of all relative averages (*p_ik_*),


$$p_{\mathrm{ik}}=\frac{n_{\mathrm{ik}}}{n_{i}\;\;n_{k}}\;\text{with}\;0\leq p_{\mathrm{ik}}\leq 1\;(\text{limiting value}\;p_{0}=0.7)$$ (4)

By using the top two interval criteria, the differential threshold is described in the following equation. Based on the average size *n*_ik_ the values of *p*_ik_ are calculated (Equation 4),


$$\lambda_{0}=\frac{2}{3}\cdot 0.7=0.683$$ (5)

The invariance matrix (*λ*) in Equation (2) serves as the complementary value against which the database for a subsequent hierarchical cluster analysis is formed. Personal groups (subgroups) that exhibit homogeneous cluster solutions of the applied concept quantities are represented by the personal clusters obtained here. To identify cluster solutions specific to each subgroup, the *Z*-matrix of the subgroup’s members was obtained by summing the individual *Z*-matrices and then standardizing the *Z*-summary values. The critical *λ*-value in Equation (5) is defined as *λ0* = 0.683 (Schack, 2002). Values equal to or above this critical *λ* threshold indicate that the knowledge storing in LTM of persons or groups are significantly similar.

## Results

Our first assumption was that experts will show a more functional structure of the represented knowledge, compared to the other participating groups. Regarding the experts’ mental representation, the SDA-M reveals four clusters (Figure 3A). The first and largest cluster contains all TRCs related to forks. The second cluster includes the skewers, the third the confinements and the fourth the pins. In comparison, intermediates’ profile (Figure 3B) shows five clusters. The first cluster contains fork-related TRCs (i.e., cluster 1.1) but the TRC *pawn fork* builds a separated cluster (i.e., cluster 1.2). Similar to the experts’ profile, the other clusters include the skewers (i.e., cluster 2), the confinements (i.e., cluster 3) and the pins (i.e., cluster 4). Moreover, Euclidean distances are smaller, indicating that experts exhibit a higher linkage between selected TRCs related to specific stimuli motifs. In other words, experts not only provide a functional clustering but also a clearer association of specific TRCs in LTM compared to intermediates and novices (for visual comparison per se, please see Figure 3). Finally, the novices’ profile displays four clusters (Figure 3C). Strikingly, only the first cluster implies TRCs that belong to the same category and the same subcategory of strategy-related patterns (i.e., absolute forks in cluster 1). Cluster 2 comprises absolute and relative pins that belong to the same category but to different subcategories. Lastly, the other clusters mix TRCs which do not belong to the same category of strategy-related patterns at all (i.e., a fork and a pin in cluster 3 and a skewer and a pin in cluster 4).

Our second assumption was that the group cluster solutions (i.e., invariance analysis) would reveal no invariance. However, the invariance comparison between experts and intermediates shows a significant similarity with *λ* = 0.837. But when comparing experts with novices, the invariance value is non-significant (*λ* = 0.575), and for intermediates versus novices, it is also non-significant (*λ* = 0.528).

## Discussion

The main aim of the present study was to gain insights into chess players’ mental representations of different categories of strategy-related patterns (i.e., TRCs). To achieve this goal, specific TRCs were chosen and the SDA-M method was applied. This method allowed us to evaluate and compare group/expertise-related mental representation structures. According to existing findings, SDA-M-based research in different domains (e.g., surfing, handball, dancing, volleyball, climbing) supports the supposition that mental representation structures of domain-specific knowledge are closely related to the expertise level of participants (Lander and Lange, 1996). We first hypothesized that, given the extensive literature on both chess expertise and human skill acquisition in cognitive psychology, experts would demonstrate more structured knowledge representations than the other groups of participants when applying SDA-M to the field of chess. Secondly, we assumed that the mental representation structures of the groups would not exhibit significant similarities.

The results fully confirm our first hypothesis. For the experts’ group, the SDA-M reveals four clusters, each reflecting one of the specific chess tactics and containing the corresponding TRCs. Additionally, the group profile for intermediates exhibits clustering similar to that of the experts, but with larger Euclidean distances (i.e., linkage distances). It is noticeable that two TRCs, which actually belong to the fork cluster, are separated by intermediates, forming a distinct pawn fork cluster. Finally, the novices’ mental representation profile shows that only some TRCs are correctly linked to each other according to the categorical criteria defined in our study.

In summary, the findings align with the supposition that the clarity and structure of mental representations are closely related to the expertise level of participants (Lander and Lange, 1996). Experts have invested more time and effort in acquiring a differentiated and precise classification of strategy-related patterns through intense training and tournament practice. As suggested by Ericsson and Lehmann (1996), Simon and Chase (1973), and Ericsson and Ward (2007), it is estimated that approximately a decade of deliberate practice is necessary to ensure successful competition in international tournaments. From this point of view, and according to the self-reported information from the chess players participating in our study, experts, although on average younger than intermediates, reported a more intensive daily training load and participated in a higher number of competitive chess events. The greater amount of deliberate practice results in the functional and effective formation of chunks, which can be translated into optimized cognitive performance.

Our second hypothesis, which stated that there would be no significant similarity between the groups’ mental representation profiles, is only partly confirmed. The invariance comparison between experts and intermediates yielded an unexpected result, showing a significant similarity. This finding may be explained by the relatively low complexity of the TRCs utilized (viz. fundamental chess-related patterns). Hence the chosen TRCs are an integral part of basic chess training. As a result, experts and intermediates might employ similar pattern recognition methods to evaluate them. Both experts and intermediates have acquired a significantly similar clustering, interpretation, and understanding of the TRC categories and subcategories. This could be explained by the fact that during intense basic training, the represented chess patterns became well-known for the participants of both groups. On the other hand, our supposition that there is no significant similarity between experts and intermediates compared to novices is confirmed. These results affirm the assumption that expertise in chess is related to the amount of deliberate practice.

In summary, our results help to identify expertise-dependent differences in chess players’ mental representation structures of strategy-related patterns. The observed differences indicate that a higher level of expertise corresponds to a more differentiated and concise classification of strategy-related patterns in LTM. The significant investment of time and effort by experts into rigorous training, tournament participation, and the retrograde analysis of high-level competitions (i.e., deliberate practice) results in a superior cognitive setting (i.e., chunking). Consequently, it can be assumed that expertise-dependent chunking leads to a reduction in cognitive costs and facilitates the retrieval of chess knowledge (Chase and Simon, 1973a; Gobet and Simon, 1996; Simon and Gilmartin, 1973).

On the other hand, the mental representation profile of intermediates reveals structures similar to those of the experts. However, a closer examination of the Euclidean distances shows that the TRCs are less interconnected compared to the experts’ cluster solution. This phenomenon can be attributed to the relatively lower time investment in chess activities (e.g., training and deliberate practice). As expected, novices exhibit a less pronounced classification of TRCs compared to the other groups. The analysis revealed no significant similarities, which aligns with previous findings in other domains (Velentzas et al., 2011).

According to the results, we can assume that evaluating tactical knowledge in chess can be helpful in identifying weaknesses and subsequently planning specific interventions. Furthermore, we suggest that chess players should extensively train their tactical understanding by explicitly identifying and categorizing strategy-related patterns in randomized scenarios (e.g., randomized chess puzzles). This approach helps to abstract and classify the vast array of critical game situations, creating a manageable taxonomy for optimal organization of chess knowledge. In turn, evaluating mental representations can be beneficial for the development and programming of virtual players (platforms, Dhou, 2018) that support and individualize chess training. This intervention can be monitored through the reassessment of SDA-M.

Further research is needed to gain deeper insights into experts’ mental representations and to clarify the transition boundaries between different performance levels. Therefore, future research should explore more complex TRCs that involve intricate subcategorizations, which result in significant differences between experts’ and intermediates’ clustering of the TRCs. Another research opportunity could be the manipulation of tactical scenarios using real game situations and simultaneously evaluating decision-making accuracy, decision-making time, and/or analyzing visual search processes.

## Limitations

The present study is primarily limited by the restricted set of TRCs employed. It is acknowledged that the scope of the examination could be expanded in the future to include a wider range of TRCs, such as additional strategy-related patterns. This would enhance the understanding of how TRCs are clustered in the mental representations of chess players with different skill levels. Furthermore, a more comprehensive SDA-M-based evaluation of players’ hierarchical chess TRC clustering in their mental representations may facilitate the identification of transitional boundaries between different skill levels (e.g., from novice to intermediate). However, it is important to acknowledge the practical constraints on the number of TRCs that can be utilized in a single split procedure.

Secondly, both the relatively small sample size (*n* < 20 per expertise group) and the fact that all participants belong to the same cultural/geographical background (i.e., middle/northern Germany) limit the generalizability of the results. This also provides an impetus to further develop the experimental method to be semi-automated and conducted online.

## Data Availability

The raw data supporting the conclusions of this article will be made available by the authors, without undue reservation.

## Footnotes

1 DWZ = *Deutsche Wertungszahl* – *German rating system* which corresponds to the international ELO.

2 While *λ* denotes a scalar, we use the notation (*λ*) to represent the matrix (*λ*_ik_)_1 ≤ i, *k* ≤ *N*_.

## References

[ref1] Amidzic O. Riehle H. J. Fehr T. Wienbruch C. Elbert T. (2001). Pattern of focal γ-bursts in chess players. Nature 412, 603–603. doi: 10.1038/35088119, 11493907

[ref2] Anderson R. C. (1977). “The notion of schemata and the educational enterprise: general discussion of the conference” in Schooling and the acquisition of knowledge. eds. Anderson R. C. Spiro R. J. Montague W. E. (Hillsdale, NJ: Erlbaum), 415–432.

[ref3] Barking M. (2024). Language learners, chess champions, and piano prodigies–insights from research on language contact and expert behavior. Yearb. Ger. Cogn. Ling. Assoc. 12, 259–288. doi: 10.1515/gcla-2024-0011

[ref4] Bilalić M. Kiesel A. Pohl C. Erb M. Grodd W. (2011). It takes two-skilled recognition of objects engages lateral areas in both hemispheres. PLoS One 6:e16202. doi: 10.1371/journal.pone.0016202, 21283683 PMC3025982

[ref5] Bläsing B. Güldenpenning I. Koester D. Schack T. (2014). Expertise affects representation structure and categorical activation of grasp postures in climbing. Front. Psychol. 5:1008. doi: 10.3389/fpsyg.2014.01008, 25309480 PMC4164095

[ref6] Bläsing B. Tenenbaum G. Schack T. (2009). The cognitive structure of movements in classical dance. Psychol. Sport Exerc. 10, 350–360. doi: 10.1016/j.psychsport.2008.10.001

[ref7] Braun S. M. Beurskens A. J. Schack T. Marcellis R. G. Oti K. C. Schols J. M. . (2007). Is it possible to use the structural dimension analysis of motor memory (SDA-M) to investigate representations of motor actions in stroke patients? Clin. Rehabil. 21, 822–832. doi: 10.1177/0269215507078303, 17875562

[ref8] Chase W. G. Simon H. A. (1973a). “The mind’s eye in chess” in Visual information processing. ed. Chase W. G. (New York: Academic Press), 215–281.

[ref9] Chase W. G. Simon H. A. (1973b). Perception in chess. Cogn. Psychol. 4, 55–81. doi: 10.1016/0010-0285(73)90004-2

[ref10] Colditz K. (2016). Lehr-, Übungs- und Testbuch der Schachkombinationen (14th ed). Oetwil, Switzerland: Edition Olms. 9783283010270.

[ref11] Cowan N. (2008). What are the differences between long-term, short-term, and working memory? Prog. Brain Res. 169, 323–338. doi: 10.1016/S0079-6123(07)00020-9, 18394484 PMC2657600

[ref12] De Groot A. D. (1946/1966). Het Denken van den Schaker [Thought and Choice in Chess]. New York: Basic Books Original work published 1946.

[ref13] Dhou K. (2018). “Towards a better understanding of chess players’ personalities: a study using virtual chess players” in Human-computer interaction. Interaction technologies. HCI 2018 (Lecture Notes in Computer Science. ed. Kurosu M., vol. 10903 (Cham: Springer).

[ref14] D’iakov (1927). Psychologie des Schachspiels: auf der Grundlage psychotechnischer Experimente an den Teilnehmern des Internationalen Schachturniers zu Moskau 1925. Berlin: W. de Gruyter & co.

[ref15] Elo A. (1978). The rating of chess players, past and present. New York, NY: Arco.

[ref16] Ericsson K. A. Lehmann A. (1996). Expert and exceptional performance: evidence of maximal adaptation to task constraints. Annu. Rev. Psychol. 47, 273–305. doi: 10.1146/annurev.psych.47.1.273, 15012483

[ref17] Ericsson K. A. Ward P. (2007). Capturing the naturally occurring superior performance of experts in the laboratory: toward a science of expert and exceptional performance. Curr. Dir. Psychol. Sci. 16, 346–350. doi: 10.1037/1076-898X.13.3.115

[ref18] Eysenck M. W. Keane M. T. (2005). Cognitive psychology. A students handbook. New York: Psychology Press.

[ref20] Gobet F. Simon H. A. (1996). Recall of random and distorted chess positions: implications for the theory of expertise. Mem. Cogn. 24, 493–503. doi: 10.3758/BF03200937, 8757497

[ref21] Hennig L. Ghesneh M. Mack M. Heinen T. (2017). Development of individual instructions based on pupils' mental representations of a gymnastics skill. J. Phys. Educ. Sport 17, 2604–2611. doi: 10.7752/jpes.2017.04297

[ref22] Krogius N. Taimanov M. Livshits A. Parma B. (1980). Encyclopedia of chess middle games: combinations. Berlin: Chess Informant.

[ref24] Lander H. J. Lange K. (1996). Untersuchung zur Struktur- und Dimensionsanalyse begrifflich-repräsentierten Wissens. Z. Psychol. 204, 55–74.

[ref25] Miller G. A. Galanter E. Pribram K. H. (1960). Plans and the structure of behavior. New York City, NYC: Henry Holt and Co.

[ref26] Miyake A. Shah P. (1999). Models of working memory: mechanisms of active maintenance and executive control. New York, NY: Cambridge University Press.

[ref27] Neistadt J. (1997). Zauberwelt der Kombinationen: Berlin: Ullstein: Taschenbuchverlag.

[ref28] Nichelli P. Grafman J. Pietrini P. Alway D. Carton J. C. Miletich R. (1994). Brain activity in chess playing. Nature 369, 191–191. doi: 10.1038/369191a0, 8183339

[ref29] Onofrj M. Curatola L. Valentini G. Antonelli M. Thomas A. Fulgente T. (1995). Non-dominant dorsal-prefrontal activation during chess problem solution evidenced by single photon emission computerized tomography (SPECT). Neurosci. Lett. 198, 169–172. doi: 10.1016/0304-3940(95)11985-6, 8552313

[ref31] Sackett R. S. (1934). The influences of symbolic rehearsal upon the retention of a maze habit. J. Gen. Psychol. 10, 376–395. doi: 10.1080/00221309.1934.9917742

[ref32] Schack T. (2002). Zur kognitiven Architektur von Bewegungshandlungen –modelltheoretischer Zugang und experimentelle Untersuchungen. Unveröff. Habil., Psychologisches Institut, Cologne: Deutsche Sporthochschule, Köln

[ref33] Schack T. (2004). The cognitive architecture of complex movement - new perspective in movement science. Special issue part II: representation and planning. Int. J. Sport Exerc. Psychol. 2, 403–438. doi: 10.1080/1612197X.2004.9671753

[ref34] Schack T. (2010). Die kognitive architektur menschlicher bewegungen: innovative zugänge für psychologie, sportwissenschaft und robotik: Aachen: Meyer & Meyer Verlag.

[ref35] Schack T. (2012). “Measuring mental representations” in Handbook of measurement in sport and exercise psychology. (Eds.) Gershon, T., Robert, C. E., and Akihito, K. 203–214. Champaign, IL, USA.

[ref36] Schack T. Hackfort D. (2007). “Action-theory approach to applied sport psychology” in Handbook of sport psychology. eds. Tenenbaum C. Eklund R. C.. 3rd ed (New York: Wiley), 332–351.

[ref37] Schack T. Mechsner F. (2006). Representation of motor skills in human long-term memory. Neurosci. Lett. 391, 77–81. doi: 10.1016/j.neulet.2005.10.009, 16266782

[ref38] Schack T. Tenenbaum G. (2004). Perceptual and cognitive control in action–a preface. Int. J. Sport Exerc. Psychol. 2, 207–209. doi: 10.1080/1612197X.2004.9671742

[ref40] Simon H. A. (1996). The sciences of the artificial. Cambridge, MA: MIT Press isbn:9780262193740.

[ref41] Simon H. A. Chase W. G. (1973). Skill in chess. Am. Sci. 61, 394–403.

[ref42] Simon H. A. Gilmartin K. J. (1973). A simulation of memory for chess positions. Cogn. Psychol. 5, 29–46. doi: 10.1016/0010-0285(73)90024-8

[ref43] Strenge B. Schack T. (2021). Empirical relationships between algorithmic SDA-M-based memory assessments and human errors in manual assembly tasks. Sci. Rep. 11:9473 (2021). doi: 10.1038/s41598-021-88921-1, 33947879 PMC8097020

[ref44] Tarrasch S. (1931). Das Schachspiel. Darmstadt: Deutsche Buch-Gemeinschaft C. A. Koch’s Verlag.

[ref45] Thomas K. T. Thomas J. R. (1994). Developing expertise in sport: the relation of knowledge and performance. Int. J. Sport Psychol. 25, 295–295.

[ref46] Velentzas K. Heinen T. Schack T. (2011). Routine integration strategies and their effects on volleyball overhand serve performance and mental representations. J. Appl. Sport Psychol. 23, 209–222. doi: 10.1080/10413200.2010.546826

[ref47] Velentzas K. Heinen T. Tenenbaum G. Schack T. (2010). Functional mental representation of volleyball routines in German youth female national players. J. Appl. Sport Psychol. 22, 474–485. doi: 10.1080/10413200.2010.504650

[ref48] Vogel L. Schack T. (2023). Cognitive representations of handball tactic actions in athletes–the function of expertise and age. PLoS One 18:e0284941. doi: 10.1371/journal.pone.0284941, 37141271 PMC10162645

